# The Mechanism, Clinical Efficacy, Safety, and Dosage Regimen of Atomoxetine for ADHD Therapy in Children: A Narrative Review

**DOI:** 10.3389/fpsyt.2021.780921

**Published:** 2022-02-09

**Authors:** Di Fu, Dan-Dan Wu, Hong-Li Guo, Ya-Hui Hu, Ying Xia, Xing Ji, Wei-Rong Fang, Yun-Man Li, Jing Xu, Feng Chen, Qian-Qi Liu

**Affiliations:** ^1^Department of Pharmacy, Pharmaceutical Sciences Research Center, Children's Hospital of Nanjing Medical University, Nanjing, China; ^2^School of Basic Medical Sciences and Clinical Pharmacy, China Pharmaceutical University, Nanjing, China; ^3^Department of Children Health Care, Children's Hospital of Nanjing Medical University, Nanjing, China

**Keywords:** atomoxetine, ADHD, children, mechanism of action, clinical efficacy, dosage, adverse reactions

## Abstract

Atomoxetine, a selective norepinephrine (NE) reuptake inhibitor, was approved for attention deficit/hyperactivity disorder (ADHD) treatment in children, adolescents and adults. We searched the database PubMed/MEDLINE (2000 to October 1, 2021). Only publications in English were considered. Atomoxetine inhibits the presynaptic norepinephrine transporter (NET), preventing the reuptake of NE throughout the brain along with inhibiting the reuptake of dopamine in specific brain regions such as the prefrontal cortex (PFC). The novel mechanism of atomoxetine also includes several new brain imaging studies and animal model studies. It is mainly metabolized by the highly polymorphic drug metabolizing enzyme cytochrome P450 2D6 (CYP2D6). Atomoxetine is effective and generally well tolerated. ADHD is often accompanied by multiple comorbidities. A series of studies have been published suggesting that atomoxetine is effective in the treatment of ADHD symptoms for children with various types of comorbidity. In some cases, it is possible that atomoxetine may have a positive influence on the symptoms of comorbidities. Atomoxetine can be administered either as a single daily dose or split into two evenly divided doses, and has a negligible risk of abuse or misuse. The latest guideline updated that clinical dose selection of atomoxetine was recommended based on both *CYP2D6* genotype and the peak concentration. To have a more comprehensive understanding of atomoxetine, this review sets the focus on the mechanism, clinical efficacy and dosage regimen in detail, and also touches on those studies regarding adverse reactions of atomoxetine.

## Introduction

ADHD is a neuropsychiatric disorder commonly diagnosed in children and adolescents. It affects their learning, emotion, cognition, and social behavior to varying degrees (1, 2). The pooled worldwide ADHD prevalence among children varies from 2 to 18% (3). The prevalence in Chinese children and adolescents can reach 5.6–8.4% (4), and there is a significant sex disparity in the prevalence (14% in boys and 6.3% in girls) (5). The core symptoms of ADHD include impaired levels of inattention, hyperactivity, or impulsivity, or a combination (6). ADHD is associated with neurochemical signaling dysregulations in several brain regions, particularly in the PFC, involving attention and executive functions (7, 8). Through dopamine and NE signaling mechanisms, brainstem neuronal projections connect to the PFC to regulate behavior (7, 9). There is growing evidence that the input of catecholamine in the PFC of patients with ADHD is reduced (8). Magnetic resonance imaging (MRI) studies have demonstrated that children with ADHD have slightly smaller, on average, entire brain and decreases in certain cortical and subcortical regions (10). Functional MRI (fMRI) is an emerging neuroimaging technique widely used to measure brain activity *in vivo* (11, 12). Compared with the control group, children with ADHD have decreased activation in the inferior frontal cortex (IFC), dorsolateral prefrontal cortex (DLPFC), insula, striatum, thalamic and parietal regions, as well as cerebellum, and increased activation in regions of the default mode network (DMN) in fMRI studies of attention functions (13–15). Additionally, comorbid psychiatric disorders are common among child patients with ADHD (16).

The commonly used treatment methods for ADHD include pharmacological treatment (17, 18), behavioral therapy (19, 20), combined medication and behavior therapy (21, 22). Pharmacological treatments for ADHD include stimulants (e.g., methylphenidate and amphetamines) generally recommended as the first-line treatment (23) and non-stimulants (e.g., alpha 2 agonists and atomoxetine) (24) considered to be the second-line treatment (3, 18). In contrast to European and North American guidelines, the 2014 Japanese clinical guidelines and the 2015 Chinese guideline recommend both non-stimulants and stimulants as the first-line pharmacological treatment for children and adolescents with ADHD. Atomoxetine is the first non-stimulant medication which was approved by the US Food and Drug Administration (FDA) to treat ADHD in late 2002 (25), and typically is prescribed to children who cannot tolerate or do not respond to stimulants (26). It generally takes 2–4 weeks for full impact on symptoms to be observed (27). Atomoxetine is generally considered to be safe and effective, and its use is associated with relatively few adverse reactions (1, 28). It is mainly metabolized by the highly polymorphic drug metabolizing enzyme CYP2D6, which has significant genetic polymorphism (29). The metabolic ability of CYP2D6 enzymes to atomoxetine is significantly different among different individuals, affecting the therapeutic efficacy and dosage regimen of clinical treatment of ADHD (29, 30).

This review focuses on the mechanism, clinical efficacy, dosage regimen, and adverse reactions of atomoxetine. Regarding the mechanism of atomoxetine, the known studies about norepinephrine reuptake inhibitor (NRI) and several new brain imaging studies were reported. The clinical efficacy was described through three aspects: short-term efficacy, long-term efficacy, and comorbidities. The reasons for the better efficacy in older children than in younger children were also discussed. In the description of dosage, not only the standard dosing regimen, but also *CYP2D6* genotype and the peak concentration recommended in the latest guideline are included to guide the clinical atomoxetine dosage selection. In addition, the influence of administration time (in the morning or at night), administration frequency and administration rate on clinical efficacy was also discussed. Common adverse reactions include the latest research findings on somnolence and nighttime awakening.

## Methods

We searched the database PubMed/MEDLINE (January 1, 2000 to October 1, 2021). Searches included keywords (Medical Subject Heading) and free-text terms for ADHD, atomoxetine, children, mechanism, brain, clinical efficacy, short-term efficacy, long-term efficacy, dosage, comorbidities, oppositional defiant disorder (ODD), anxiety, autism spectrum disorder (ASD), tics disorders (TD), Tourette's Syndrome (TS), learning disorders, adverse reactions, sleep, older, and safety. Only articles in English were reviewed to determine suitability for inclusion.

## Mechanism of Action

**Figure 1:**
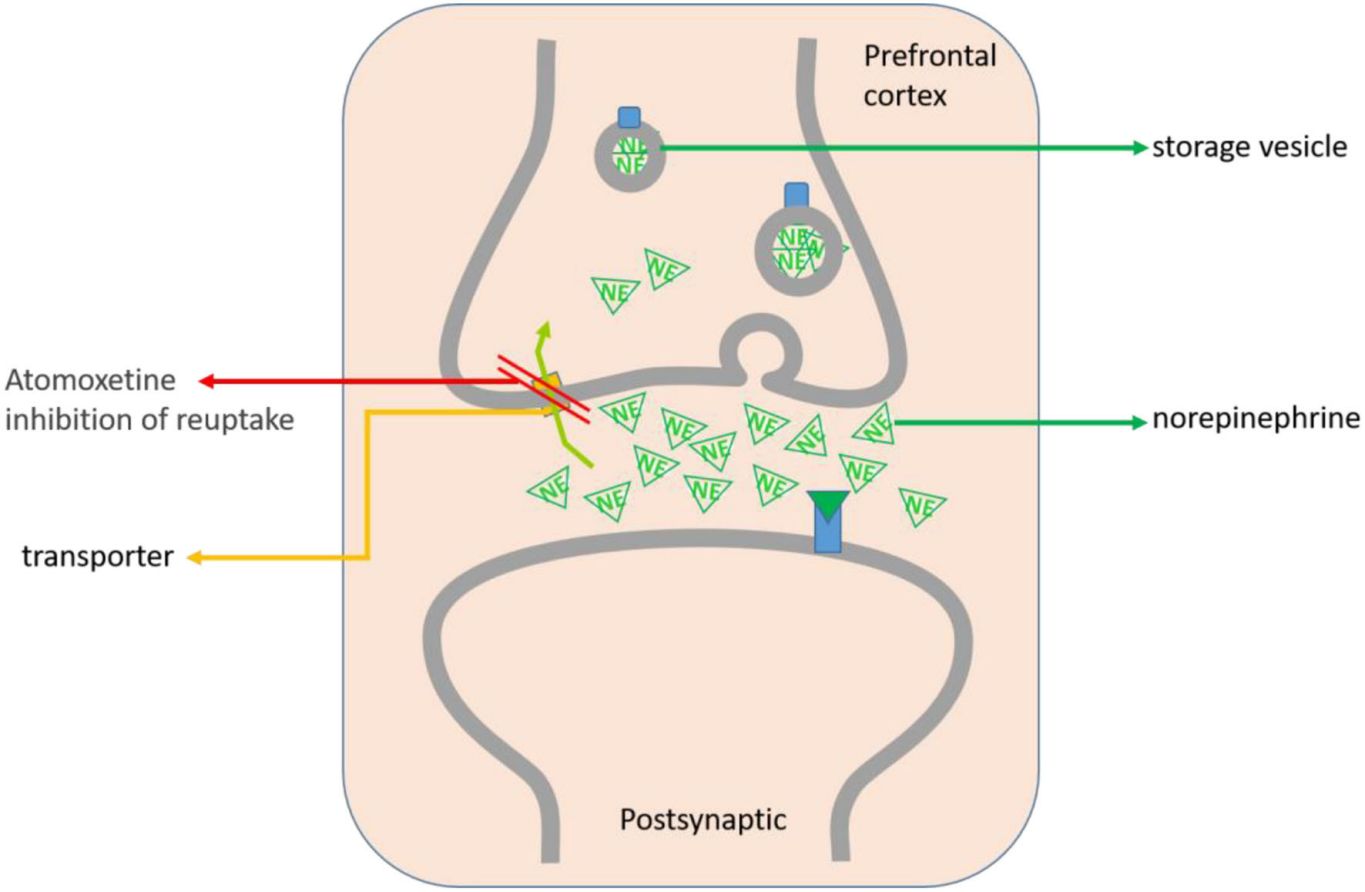
The MOA of atomoxetine on ADHD treatment is mainly located in the PFC. Atomoxetine can highly bind to the NE reuptake transporter on the presynaptic membrane of the PFC, selectively inhibit NE reuptake and increase the NE in the synaptic cleft of neurons. Atomoxetine can also modulatethe dopamine uptake in the synaptic cortex through NET, and increase the dopamine level in the PFC, but not in the nucleus accumbens and striatum.

While the precise mechanism of action (MOA) through which atomoxetine produces its therapeutic effects is unclear, the putative hypotheses have been elucidated (28, 31). Atomoxetine is thought to be related to its selective inhibition of presynaptic NE reuptake in the PFC (32). As a highly selective norepinephrine reuptake inhibitor (SNRI), atomoxetine highly binds to NE reuptake transporter on the presynaptic membrane of the nerve and inhibits NE reuptake (32). Its role is to increase the NE concentration in the synaptic cleft, which results in elevations of synaptic levels of noradrenaline in the central nervous system (1, 33, 34). However, it has little or no affinity for either the serotonin or dopamine transporters or other neurotransmitter receptors (35). Additionally, atomoxetine is also known to modulate cortical synaptic dopamine uptake via the nonspecific action of the abundant NET in the PFC, selectively increasing dopamine levels in the PFC in a regionally specific manner (27, 36), but not in motor or reward-related areas of the striatum (34), thereby improving the symptoms of ADHD and concentration of attention (Figure 1).

As suggested by Barr et al. (37), the pathogenesis of ADHD is related to the dopamine decrease and the NE reversal effect. This finding is consistent with the pharmacological effects, which theoretically explains the effectiveness in the treatment of ADHD. Atomoxetine exerts a reversal effect by gradually increasing extracellular levels of NE. One rat study (32) found that atomoxetine showed a high affinity for the NET, a low affinity for the serotonin transporter, and a relatively low affinity for dopamine transporter. The high affinity for the NET prevented the reuptake of norepinephrine, leading to an increase in neurotransmitters at the neuronal synapses in the PFC of the rat brain.

Fortunately the progress of human brain imaging studies in recent years has provided us with new insights into the ADHD management with atomoxetine (10, 13, 38–40). The right inferior PFC is a key area of dysfunction in ADHD during inhibitory performance (41). Atomoxetine has beneficial effects on inhibitory control by regulating right inferior frontal function (38). These findings of the study provided important neurofunctional evidence for the MOAs (38). Moreover, a study provided the first fractional amplitude of low-frequency fluctuations (fALFF) evidence of distinct therapeutic mechanisms of atomoxetine and methylphenidate in the treatment of children with ADHD (39). Increased fALFF in the left lingual gyrus and inferior occipital gyrus was correlated with inattention improvement with the treatment of atomoxetine. In contrast, improvement in hyperactivity/impulsivity was associated with decreased fALFF in bilateral precentral and postcentral gyri in bilateral precentral and postcentral gyri (39). The fALFF effect of atomoxetine treatment associated with clinical improvement emphasized the importance of using resting-state functional MRI (RS-fMRI) techniques to exploring the medication effects on intrinsic brain activity in children with ADHD. Children ages 7–12 years with ADHD who respond clinically to atomoxetine had improvements of clinical symptoms, correlated with a decrease in motor cortex short interval cortical inhibition (40). A randomized, double-blind, placebo-controlled, crossover study (13) shows that atomoxetine upregulates inferior frontal activation and improves DMN deactivation during cognitive functions, improves attention functions and upregulates abnormal fronto-cortical activation during executive function tasks in ADHD patients, and appears to have drug-specific effects of up-regulating DLPFC. Most importantly, this study shows for the first time that during the period of sustained attention, a single dose of atomoxetine has a comparable effect to methylphenidate in normalizing fronto-striato-thalamo-parietal brain abnormalities in ADHD (10, 13). Actually, the first study that incorporated RS-fMRI into the randomized controlled trial (RCT) of atomoxetine in medication-naive adults with ADHD suggested an important mechanism of atomoxetine therapy (42). The results showed that atomoxetine treatment caused a strengthened anti-correlated relationship between DMN and the task-positive networks (cognitive control and dorsal attention networks) (42). Also, this study for the first time found that the strengthened anti-correlations were linked to the improved ADHD symptoms after the atomoxetine therapy (42). Although this clinical trial was conducted in adults, in this way, a new possibility opens up in this area of research. So it will be very intriguing to perform a similar study in children.

A 2014 study using positron emission tomography (PET) imaging to assess clinically relevant doses of atomoxetine in rhesus monkeys found that atomoxetine occupied not only NET, but also the serotonin transporters (43). A murine prenatal nicotine exposure model of ADHD was made to investigate the effects of atomoxetine on behavior and synaptic plasticity in the hippocampus and long-term potentiation (LTP) in hippocampal slices at the CA3–CA1 synapses (44). The results indicated that atomoxetine in this model reconstructed LTP in CA3–CA1 synapses. Postsynaptic changes in synaptic plasticity may be part of the mechanisms of atomoxetine-induced improvement in ADHD symptoms (44). These findings suggest that there may be one or more unknown pathways through which atomoxetine works. The neuropharmacology of these aspects awaits further study.

## Clinical Efficacy

Clinically meaningful improvements in the core symptoms of ADHD (inattention, impulsivity, and hyperactivity) as well as quality of life and emotional lability (34) are often observed after atomoxetine therapy. The focus of this section is on data from fully published, randomized, controlled trials investigating the therapeutic effectiveness of atomoxetine in children with ADHD (Table 1). Clinical efficacy data for ADHD pediatric patients were pooled from short-term (6–16 weeks) studies (45–48, 52, 53) and long-term (>6 months) studies (49–51).

**Table 1:** Summary of efficacy trials in atomoxetine treatment studies.

	**Study (year)**	**Type of study**	**Ages (years)**	**Number of cases**	**Time**	**Groups**	**Dose**	**Comparison of ADHD-RS-IV**	**Clinical outcomes**	**References**
**Short-term treatment**	Buitelaar et al. (2004)	Prospective, Multicenter, open-label	6–15	604	10 weeks	Atomoxetine	A maximum of 1.8 mg/kg/day twice-daily	↓56.7%	↓ADHD symptoms ↑psychosocial outcomes ↑ functional outcomes	(45)
	Michelson et al. (2001)	Randomized, Placebo–controlled	8–18	297	8 weeks	Placebo Atomoxetine	0.5 mg/kg/day; 1.2 mg/kg/day; 1.8 mg/kg/day	38.3 vs. 40.2; 39.2; 39.7	↓ADHD symptoms ↑Atomoxetine 1.2 mg/kg/day and 1.8 mg/kg/day had superior outcomes. ↑Social and family functioning	(46)
	Griffiths et al. (2018)	Randomize, Double-blind, Placebo–controlled, Crossover	6–17	136	8 weeks	Placebo Atomoxetine	1.35 mg/kg	33.57 vs. 29.14	↓ADHD and anxiety symptoms no in sustained attention ↑primary cognitive outcomes of response inhibition and fear identification	(47)
	Kratochvil et al. (2011)	Double-blind, Placebo–controlled Randomized	5–6	101	8 weeks	Placebo Atomoxetine	A maximum of 1.8 mg/kg/day	Parent total ↓5.8 vs. 13.2 (*p* = 0.009) teacher total ↓5.0 vs. 12.5 (*p* = 0.02)	↓core ADHD symptoms 40% of children met response criteria compared with 22% of children on placebo (*p* = 0.1).	(48)
**Long-term treatment**	Michelson et al. (2004)	Randomized, Double-blind, Placebo–controlled	6–15	416	9 months	Placebo Atomoxetine	A maximum of 1.8 mg/kg/day	12.3 vs. 6.8	Atomoxetine was superior to placebo in preventing relapse. ↑superior psychosocial functioning	(49)
	Newcorn et al. (2006)	Randomized, Double–blind	6–16	516	8 months	Atomoxetine	0.5 mg/kg/day 1.8 mg/kg/day	↑3.1 vs. 1.1	Lower doses could be effective during maintenance treatment. ↑Reports of affective lability ↓heart rate	(50)
	Buitelaar et al. (2007)	Randomized, Double–blind	6–15	163	1 year	Placebo Atomoxetine	A maximum of 1.8 mg/kg/day	7.8 vs. 1.7 (*p* < 0.001)	Atomoxetine was superior to placebo in preventing relapse (*p* = 0.008) and in maintaining symptom response (*p* < 0.001).	(51)

*ADHD-RS-IV, Attention-Deficit/Hyperactivity Disorder Rating Scale-IV; ADHD, Attention-deficit/hyperactivity disorder*.

*Data are expressed as the mean, unless otherwise specified*.

### Short-Term Treatment

Clinically meaningful improvements in ADHD symptoms are often observed after a few weeks of atomoxetine treatment (27, 45–47, 54). For instance, in a 10-week open-label study of 604 pediatric patients with ADHD (45), ADHD-Rating Scale (ADHD-RS) total scores decreased by 56.7%, such that 69% of patients were rated as having no or minimal symptoms. Significant improvement was also observed in psychosocial and functional outcomes. A randomized, placebo-controlled, dose-response trial of 8-week duration investigated the efficacy of 3 doses of atomoxetine compared with placebo in children and adolescents with ADHD (46). Of the 381 patients screened, 297 children and adolescents were randomized to placebo or atomoxetine. The atomoxetine groups had superior outcomes in ADHD symptoms compared with placebo group. Social and family functions were also significantly improved in the atomoxetine groups. Schwartz and colleagues (54) comprehensively evaluated the efficacy and safety of atomoxetine in pediatric ADHD patients. This largest and most comprehensive meta-analysis including 3,928 pediatric patients found atomoxetine to be superior to placebo for ADHD on a number of relevant outcomes. The results showed that the short-term atomoxetine treatment can significantly improve the core symptoms. As objectively measured by performance-based tests, response inhibition and sustained attention deficit are among the most common cognitive deficits associated with ADHD (55, 56). In a double-blind, randomized, placebo-controlled crossover trial (47), atomoxetine treatment resulted in moderate improvement in response inhibition but no significant improvement in sustained attention. It improved identification of fearful faces, particularly in younger children. The effect size of improvement in ADHD symptoms was lower than that found in a meta-analysis of previous studies (−0.35 relative to −0.64) (54). The growing studies confirm that short-term atomoxetine treatment significantly reduces core symptoms of ADHD.

Between children and adolescents, there seems to be no difference in the efficacy of atomoxetine, but it is indeed more effective among older children than younger children (57, 58). An earlier meta-analysis of six clinical trials of 6–9 weeks duration compared atomoxetine efficacy vs. placebo among young children [(6–7 years; *n* = 184 atomoxetine, and *n* = 96 placebo) vs. older children (8–12 years; *n* = 544 atomoxetine, and *n* = 316 placebo)] (52). It found that, while atomoxetine was superior to placebo for mean (SD) change in both age categories in ADHD-RS scores and response rates (*p* < 0.05), older children had significantly (*p* < 0.05) greater improvements in ADHD-RS scores than younger children, regardless of whether they were receiving atomoxetine or placebo (52). Moreover, atomoxetine was also evaluated in young children (5–7 years old) (26). A total of 93 subjects were dosed in an 8-week, double-blind, placebo-controlled randomized clinical trial (48). Subjects who received atomoxetine had significantly greater improvement in ADHD-RS-IV scores than patients who received placebo. This is the first RCT of atomoxetine in children as young as 5 years (48). Common medications used for ADHD also include stimulants, such as methylphenidate and osmotic release oral system methylphenidate (OROS MPH). Methylphenidate is traditionally recognized as the first-line treatment for ADHD (59). Efficacy of both methylphenidate and atomoxetine has been established in placebo-controlled trials (60). While atomoxetine has been shown to be superior to placebo, definitive evidence suggesting comparable efficacy to psychostimulants does not exist at this time (61). Several comparative efficacy studies conducted in American and European populations suggested that, compared with atomoxetine, OROS MPH resulted in greater reduction in ADHD symptoms (53, 62, 63). Kemner et al. found that methylphenidate (*n* = 378) and atomoxetine (*n* = 139) produced similar symptom rating scale score reductions at weeks 1 and 2 in the treatment-naive subgroup (*n* = 517) in a multicenter, randomized, prospective, open-label study in 1323 American children 6 to 12 years of age with ADHD (62). Subsequently, Newcorn et al. also conducted a large placebo-controlled, double-blind, randomized study for 6 weeks in America. It also found that the response rates for OROS MPH and atomoxetine were not significantly different, but that they were markedly superior to the rate for placebo in the treatment-naive subgroup at the end-point (53). In a 24-week, open-label, head-to-head clinical trial, children and adolescents randomly assigned to OROS MPH (*n* = 80) and atomoxetine (*n* = 80). Although as early as 2 weeks after randomization, primary efficacy measure improved in both treatment groups, the ADHD-RS-IV Inattention score of the OROS MPH group was significantly lower than that of the atomoxetine group (Cohen's d, 0.46; *p* = 0.004) at week 2. However, from week 4 through week 24, there was no significant difference in response to these two medications (64). Consequently, long-term studies are needed to evaluate changes in the relative efficacy of OROS MPH and atomoxetine (64). Additionally, a study suggested that the short-term efficacy of OROS MPH and atomoxetine was similar in China (65). Both of them were effective in reducing ADHD symptoms of Chinese children and adolescents. The results showed that percentages achieving remission, robust improvement, and improvement were comparable for OROS MPH and atomoxetine treatment (35.3 vs. 37.1%, 45.4 vs. 44.8%, 65.5 vs. 66.4%).

There appears to be some heterogeneity in response rates in geographic regions (27), although atomoxetine is an effective treatment in all locations (Asia, Europe, North America, and Russia) (66), and responses of ethnic groups (i.e., Caucasians, African Americans, and Latinos) in the America seem to be similar (67, 68).

### Long-Term Treatment

Similar to the findings in short-term studies, atomoxetine remained efficacious in the long-term treatment of ADHD in children (35). Atomoxetine has been demonstrated to be effective through to the end of 8- to 24-month treatment periods (69). Thereafter, response to treatment was maintained over the ensuing period, out to 24 months after the initiation of treatment (70). However, few placebo-controlled, long-term studies of efficacy have been reported.

With respect to long-term treatment, two meta-analytic reviews of 13 trials each, one in 219 adolescents (aged 12–18 years) (70) and one in 97 young children (aged 6–7 years) (71) with ADHD who were treated with atomoxetine for at least 2 years, showed that atomoxetine retained significant (*p* < 0.001) improvement at endpoint vs. baseline in ADHD-RS scores with continuous drug intake. Data from 6- and 7-year-old children (*n* = 192) suggest that the effectiveness of atomoxetine treatment is maintained over 12–24 months (71). In this 13-study meta-analysis, the corresponding decrease in ADHD Rating Scale-IV Parent Version, Investigator Administered (ADHD – RS – IV Parent: Inv) total scores showed that there was a marked improvement in ADHD symptoms after 3 months of atomoxetine treatment, which was maintained for a minimum of 24 months in the 97 pediatric subjects who continued the atomoxetine treatment for at least 2 years (71).

In addition, atomoxetine was superior to placebo in maintaining response in a 9-month relapse prevention study (49). The results supported the value of maintenance treatment with atomoxetine in pediatric patients with ADHD who responded to initial treatment (49). Newcorn et al. (50) demonstrated that low-dose atomoxetine was an effective choice for retaining the treatment response during maintenance treatment in an 8-month, randomized, double-blind trial.

In two clinical studies with initial treatment periods of 10–52 weeks, atomoxetine has also been proved to be effective at preventing relapse of ADHD symptoms (49, 51). Buitelaar et al. (51) reported that atomoxetine was superior to placebo at preventing relapse and maintaining efficacy in children and adolescents (*n* = 163) treated with atomoxetine for 1 year, who were then randomized either to atomoxetine or placebo for a further 6 months. Many patients might stay in good condition for several months without atomoxetine therapy, after 1 year of atomoxetine therapy. As a matter of fact, in the 1 - year study, for the patients who were assigned to discontinue atomoxetine therapy, they were generally less severe than at study entry when symptoms returned (51).

### In Patients With Comorbid Conditions

Comorbid psychiatric disorders are common among patients with ADHD, including ODD, anxiety and depression disorders, ASD, TD, learning disorders (LD), disruptive behavior disorders (DBDs), and TS (16, 35, 72, 73). More than half of children and adolescents with ADHD have a comorbid condition, and more than a quarter had two or more comorbidities (16, 35). In particular, ODD/conduct disorder (CD) is among the most common comorbidities with ADHD, affected approximately 50 % of children with ADHD (46, 74–76). Up to now, a series of published studies suggested that atomoxetine is effective in the treatment of ADHD symptoms for children with various types of comorbidity (27, 74, 77–83). In some cases, it is possible that atomoxetine may have a positive influence on the symptoms of comorbidities (27, 73, 82). Detailed information about these studies is summarized in Table 2.

**Table 2:** Efficacy of atomoxetine treatment in comorbidities of ADHD.

**Comorbidities**	**Study (year)**	**Type of study**	**Number of patients**	**Ages (years)**	**Time**	**Dose**	**Clinical outcomes**	**References**
**ODD**	Newcorn JH et al. (2005)	Randomized, double-blind	297	8–18	8 weeks	0.5 mg/kg/day; 1.2 mg/kg/day; 1.8 mg/kg/day	↑significant improvement in ADHD, ODD, and quality-of-life measures ↑Comorbid group showed improvement compared with placebo at 1.8 mg/kg/day but not 1.2 mg/kg/day. The comorbid group may require higher doses.	(77)
	Dittmann RW et al. (2011)	RCT, double-blind	181	6−17	9 weeks	fast: 7 days 0.5 mg/kg, then 1.2 mg/kg; slow: 7 days each at 0.5 and 0.8 mg/kg, then 1.2 mg/kg.	↓significantly reduced symptoms of ODD/CD and ADHD ↑Slower atomoxetine-up-titration may be better tolerated.	(84)
	Bangs ME et al. (2008)	RCT	226	6–12	8 weeks	target dose 1.2 mg/kg/day	↑statistically significant improvement in ADHD and global clinical functioning It remains uncertain whether atomoxetine exerts an enduring effect on ODD symptoms.	(78)
**Anxiety and depression**	Geller D et al. (2007)	RCT, double-blind	176	8–17	12 weeks	3 days 0.8 mg/kg/day, then 1.2 mg/kg/day	↑Mean ADHD RS-IV-PI total score improved significantly for atomoxetine relative to placebo (*p* < 0.001). ↑Mean Pediatric Anxiety Rating Scale (PARS) total score improved significantly for atomoxetine relative to placebo. ↑significant improvement in ADHD and anxiety symptoms using both clinician-rated and self-rated measures	(80)
	Bangs M et al. (2007)	RCT, double-blind	142	12–18	9 weeks	1.2 mg/kg/day 1.8 mg/kg/day	↑Mean decrease in ADHD RS-IV-PI total score was significantly greater in the atomoxetine group compared with the placebo group (*p* < 0.001). Rates of treatment-emergent mania did not differ between groups. ↑effective for ADHD in adolescents with ADHD and MDD, but with no evidence for atomoxetine of efficacy in treating MDD	(85)
	Kratochvil CJ et al. (2005)	RCT, double-blind	173	7–17	8 weeks	0.5 mg/kg/day then 0.8 mg/kg/day then 1.2 mg/kg/day	↑reductions in ADHD, depressive, and anxiety symptoms for both treatment groups (*p* < 0.001 for the relevant scale in each symptom cluster)	(86)
**TD and TS**	Spencer TJ et al. (2008)	RCT, double-blind	117	7–17	18 weeks	0.5–1.5 mg/kg/day	↑improvement in ADHD symptoms ↑significantly greater reduction of tic severity on two of three measures ↑significant increases in mean pulse rate and rates of treatment-emergent nausea, decreased appetite, and decreased body weight	(81)
	Allen AJ et al. (2005)	RCT	148	7–17	18 weeks	0.5–1.5mg/kg/day	↑greater reduction of tic severity relative to placebo ↑greater improvement on the ADHD Rating Scale total score (*p* < 0.001) and CGI severity of ADHD/psychiatric symptoms scale score (*p* = 0.015) ↑greater increases in heart rate, decreases of body weight and rates of treatment-emergent decreased appetite and nausea without exacerbate tic symptoms	(82)
**ASD**	Arnold LE et al. (2006)	RCT	16	5–15	7 weeks	0.25 mg/kg/day, then every 4–5 days; 0.3–0.4 mg/kg/day For subjects taking CYP2D6 inhibitor, 0.2–0.3 mg/kg/day, at most 1.2 mg/kg/day	↑Hyperactivity subscale of the Aberrant Behavior Checklist, atomoxetine was superior to placebo (*p* = 0.043). safe and effective for treating hyperactivity in some children with ASD	(87)
	Harfterkamp M et al. (2012)	RCT, double-blind	97	6–17	8 weeks	1.2 mg/kg/day	↑The Conners Teacher Rating Scale-Revised: Short Form (CTRS-R:S) Hyperactivity sub-score improved significantly for atomoxetine ↑moderately improved ADHD symptoms	(88)

*ODD, Oppositional defiant disorder; ADHD, Attention-deficit/hyperactivity disorder; RCT, randomized, placebo-controlled; CD, Conduct disorder; ADHD RS-IV-PI, ADHD Rating Scale-IV-Parent Version, Investigator Administered and Scored; PARS, Pediatric Anxiety Rating Scale; MDD, Major depressive disorder; TD, Tics disorders; TS, Tourette syndrome; CGI, Clinical Global Impressions; ASD, Autism spectrum disorder; CTRS-R:S, Conners Teacher Rating Scale-Revised, Short Form*.

#### Oppositional Defiant Disorder (ODD)

ODD is the most common and the most noticeable psychiatric disorder that co-exists with ADHD (73, 89, 90). Many well-designed studies indicated that ADHD symptoms were improved with atomoxetine treatment in ADHD patients with comorbid ODD (74, 77–79), several of which have also shown atomoxetine to be effective in improving ODD symptoms (73). However, it is currently uncertain whether or not the efficacy of atomoxetine as a treatment for ADHD is affected by comorbid ODD (27).

In a 9-week, randomized, placebo-controlled study in ADHD patients with comorbid ODD or CD, atomoxetine was significantly superior to placebo in reducing symptoms of ODD/CD and ADHD (*p* < 0.001) (84). Moreover, in a meta-analysis of 25 double-blind randomized controlled trials, atomoxetine was associated with somewhat less improvements in ODD symptoms (54). Newcorn et al. (77) reported on atomoxetine therapy in children and adolescents with ADHD and comorbid ODD. The results showed that atomoxetine treatment improved ADHD and ODD symptoms in youths, although the comorbid group may require higher doses (77). Currently, when ODD comorbidity is present, additional research may be warranted to evaluate whether higher doses are required. However, other studies indicate that although ADHD symptoms improved, there are no statistically significant improvements after atomoxetine therapy (78, 91).

#### Anxiety and Depression

Studies have reported that the prevalence of ADHD comorbid anxiety disorder is 5.6–37.9% (92, 93). Atomoxetine appears to be an effective treatment for ADHD in children with comorbid anxiety or depression (27, 80).

In 176 pediatric patients with ADHD and comorbid anxiety disorders, 12 weeks of atomoxetine appeared to be more efficacious as a treatment for ADHD symptoms vs. placebo (mean change from baseline −10.5 vs. −1.4; *p* < 0.001), and was accompanied by improvements in anxiety (80). Results supported consideration of atomoxetine for the treatment of ADHD in patients who have ADHD with comorbid anxiety disorder. In 142 adolescents with ADHD and comorbid major depressive disorder (MDD), improvements in ADHD symptoms were greater after 9 weeks of atomoxetine treatment vs. placebo, without improvement in MDD (85). The trial did not show any difference in improving depressive symptoms (Children's Depression Rating Scale-Revised [CDRS-R] scores −14.8 vs. −12.8 for atomoxetine vs. placebo; *p* = 0.34) (85). In some ways, atomoxetine has demonstrated a positive effect on anxiety symptoms (73). In a randomized, double-blind, placebo-controlled study (80), its efficacy was perceived by both the physicians and the pediatric patients with ADHD and anxiety disorders, and in a double-blind study comparing atomoxetine alone with atomoxetine in combination with fluoxetine, reductions in ADHD, depressive, and anxiety symptoms were marked for both treatment groups (*p* < 0.001 for the relevant scale in each symptom cluster) (86).

#### Tics Disorders (TD) and Tourette's Syndrome (TS)

Comorbidity of ADHD and TD or TS is common, with ADHD occurring in as many as 50% of all cases of TS in children (82). Atomoxetine appears to be effective in treating ADHD without aggravating in patients with both conditions (73).

Allen et al. (82) designed a clinical trial to determine whether atomoxetine was effective for ADHD symptoms in children with tics and ADHD. Patients were randomly assigned to double-blind treatment with placebo (*n* = 72) or atomoxetine (*n* = 76) for up to 18 weeks. The results indicated that atomoxetine was associated with a beneficial effect on tic severity, as atomoxetine treatment resulted in a significantly greater improvement than placebo (−0.7 vs. −0.1; *p* = 0.002) in the Clinical Global Impressions Tic/Neurologic Severity (CGI-Tic/Neuro-S) Scale scores, and both atomoxetine and placebo groups achieved significant improvements from baseline in tic severity assessed by the Yale Global Tic Severity Scale total score (YGTSS, primary measure of tic severity; −5.5 vs. −3.0) and the Tic Symptom Self-Report total score (TSSR; −4.7 vs. −2.9). Another study also has shown atomoxetine is efficacious for treatment of ADHD and well tolerated in ADHD children with comorbid TS (81).

#### Learning Disorders (LD)

The most common LD is reading disorder (RD), or dyslexia (94). Estimates of rates in those with ADHD and comorbid dyslexia range from 15 to 45%, much greater than by chance (95). A randomized, placebo-controlled trial evaluated the efficacy of atomoxetine in children with dyslexia only or ADHD and comorbid dyslexia (95).

The results showed reading scores were improved with atomoxetine therapy in children with ADHD and comorbid dyslexia. But in general, the improvements of ADHD symptoms in children with ADHD and comorbid dyslexia were not associated with improvement of reading scores (95–97). As a result, it is vital to treat ADHD and learning disorders together rather than in isolation (94). The association between atomoxetine and dyslexia-related improvements (including word recognition, comprehension, and reading ability) deserves more attention and research (72).

#### Autism Spectrum Disorder (ASD)

About 20–60% of children with ADHD have autism-like social difficulties higher than typically developing children (98). Initiation of medication in ADHD with ASD is much earlier than in ADHD children (83).

In some of studies to date, atomoxetine has been shown to be safe and effective for treating ADHD symptoms in some children with ASD (88, 99), but the response rate has been nominally less than that reported for developing children with ADHD alone (87). A small (*n* = 16), double-blind study in children with ASD and ADHD randomly assigned to order in a crossover of clinically titrated atomoxetine and placebo revealed that, compared with placebo, atomoxetine was associated with significant improvement in Aberrant Behavior Checklist—Hyperactivity (ABC-H) scores (primary endpoint) and ABC—Lethargy/Social Withdrawal scores (the ABC is a widely used rating scale for evaluating patients with developmental disabilities). On the primary outcomes, atomoxetine was superior to placebo on the hyperactivity subscale of the ABC (*p* = 0.043). ABC-H scores of the atomoxetine group decreased from 24.69 at baseline to 19.31 at week 6, and these of placebo group dropped from 22.50 at baseline to 22.37 at week 6 (*p* = 0.04), while ABC-Lethargy/Social Withdrawal scores were reduced from 8.69 to 6.50 in the atomoxetine group, but increased from 6.62 to 7.43 in the placebo group (*p* = 0.01) (87). Additionally, results of a systematic review and meta-analysis demonstrate the beneficial effect of atomoxetine therapy on ADHD symptoms in children with comorbid ASD (83). Although evidence suggests potential efficacy of atomoxetine, the existing evidences are not conclusive and more trials are needed (99, 100).

### Dosage

#### Standard Dosage of Atomoxetine

Currently, atomoxetine is only used in pediatric patients over 6 years old with ADHD (101). The efficacy and safety of atomoxetine in ADHD patients ≤ 6 years old have not been established, and its application in preschool children with ADHD is still limited. Dosage of atomoxetine in children was initially studied in an open-label and prospective trial of 30 subjects with ADHD aged 7–14 years (102). Mean final total daily dose was 1.9 mg/kg/day (range: 0.4–3.2 mg/kg/day). Atomoxetine significantly decreased core ADHD symptoms as measured by the investigator-scored ADHD Rating Scale-IV (38.6% decrease vs. baseline, *p* < 0.001) (102).

In the European Summary of Product Characteristics (SPC) and US Full Prescribing Information for atomoxetine, body weight-based dosing is recommended for children and adolescents up to 70 kg. The starting dose recommended in the published prescribing information is approximately 0.5 mg/kg/day which can then be increased to a target maintenance dose of approximately 1.2 mg/kg/day (for a minimum of 7 days in Europe, and a minimum of 3 days in the America). The maximum total daily dose approved by the FDA for children and adolescents who weigh ≤ 70 kg is 1.4 mg/kg/day; for children and adolescents who weigh > 70 kg, it is 100 mg/day (according to the product labeling). However, the European SPC states that the safety of atomoxetine dose > 1.8 mg/kg/day “have not been systematically evaluated”. For children and adolescents over 70 kg, the recommended dose is to increase from a daily initiation dose of 40–80 mg, and the maximum total daily dose should not exceed 100 mg in the USA and Europe. It is manufactured in capsules containing 10, 18, 25, 40, 60, 80, or 100 mg of atomoxetine hydrochloride as well as in an oral solution (4 mg/ml) (103).

To date, only one study assessed the utility and tolerability of higher than standard dosage to treat ADHD (104). Two randomized, double-blind trials were conducted in patients ages 6–16 years who did not respond to atomoxetine comparing continued treatment with same-dose atomoxetine treatment using greater than standard dosage (study 1: up to 3.0 mg/kg/day; study 2: up to 2.4 mg/kg/day). The results showed no improved overall response in children or adolescents who did not respond to lower doses of atomoxetine (1.2 to 1.8 mg/kg/day) (104). Higher doses do not seem to achieve better efficacy outcomes. Currently, there is no data to support increased effectiveness at higher doses.

In addition, the initial and target doses of atomoxetine should be reduced to 50% of the normal dose in patients with moderate hepatic impairment and to 25% of the normal dose in those with severe hepatic impairment (105).

The Clinical Pharmacogenetic Implementation Consortium (CPIC) updated the guidelines in February 2019 for clinical dose selection of atomoxetine based on *CYP2D6* genotype and the peak concentration (30). According to the individual's ability to metabolize drugs, individuals with different *CYP2D6* phenotypes can be divided into four categories: extensive metabolizer (EM), poor metabolizer (PM), intermediate metabolizer (IM) and ultrarapid metabolizer (UM) (106). UMs rapidly metabolize atomoxetine, so that the plasma concentration of drugs decreases too fast to achieve the ideal clinical efficacy, while PMs may lead to the accumulation in the body because of slow metabolism, increasing the risk of adverse reactions (30, 107). The recommendation is to initiate dosing at 0.5 mg/kg/ day and increase to 1.2 mg/kg/ day after 3 days in EMs and UMs, while also utilizing plasma concentrations to reach peak concentrations approaching 400 ng/mL after 2 weeks if there is no clinical response or adverse reactions. In PMs and IMs, an initial dose of 0.5 mg/kg/ day is recommended, followed by a 2-week wait, with peak plasma concentrations guiding dose adjustments in the absence of clinical response and adverse reactions (30).

#### Administration Time and Titration Rate

With regard to once- or twice-daily dosing, and morning vs. afternoon/evening dosing, similar advice is issued in the European SPC and US Full Prescribing Information document for atomoxetine. Atomoxetine should be administered as a single dose in the morning, or twice daily as evenly divided doses in the morning and late afternoon/early evening (according to the product labeling). In this section, we reviewed findings from trials comparing once- or twice-daily dosing and the effect of titration rate on therapeutic efficacy of atomoxetine therapy and provided a summary of the dosing studies.

Atomoxetine appeared to be as effective when the daily dose was administered once in the morning as when the dose was divided and administered in the morning and evening in a double-blind study (79). Both once-daily morning and evening dosage regimens have been evaluated (74, 79, 108). A 3-arm and randomized trial of morning and evening atomoxetine dosing compared to placebo in 288 children aged 6–12 years showed that ADHD-RS-IV total scores improved significantly during the 6-week trial (*p* < 0.001) for morning dosing while ADHD-RS-IV total scores with evening dosing was not significantly different from placebo. Morning- and evening-dosed atomoxetine were significantly better than placebo in the mean changes for evening behavior using the Conners' Global Index-Parent Version (CGI-P) ratings (*p* < 0.001 and *p* < 0.05, respectively). Evening dosing showed greater tolerability than morning dosing (79).

In a *post hoc* analysis of 22 pediatric and 3 adult atomoxetine trials in subjects with ADHD, the effects of a daily dosage regimen and titration rate were evaluated (109). Pediatric patients who received once-a-daily dosing had a significantly earlier onset of all treatment-emergent adverse events (TEAEs), and the fast titration method has a shorter time to onset for common TEAEs than the slow titration method (109). Slower up-titration (0.5 mg/kg/day for 7 days, followed by 0.8 mg/kg/day for 7 days, and then 1.2 mg/kg/day) may be more beneficial than faster up-titration (0.5 mg/kg/day for 7 days, then 1.2 mg/kg/day) (26, 109). For children with ADHD who have seriously decreased appetite after atomoxetine treatment, the initial dose can be changed from once daily to twice daily, fast to slow. Additionally, Dittmann et al. (84) demonstrated that slower atomoxetine-up-titration may be better tolerated than faster up-titration, although improvements did not differ significantly between both titration groups in this 9-week study of 181 children or adolescents with ADHD and comorbid ODD.

Moreover, a non-randomized, open-labeled, prospective interventional study was conducted to clarify the relationship between the plasma concentration and clinical efficacy of atomoxetine (110). Participants aged 6–17 were first administered 0.5–0.8 mg/kg of atomoxetine and the dose was increased every 2 weeks to a maximum of 1.8 mg/kg, with the exceptional cases in which adverse reactions emerged. The responder-related dose (excluding outliers) of 1.55 ± 0.28 mg/kg identified in this study may be helpful to clinicians. When titrating atomoxetine in general clinical practice, this response-related dose may be one of the targets. The minimal adverse reactions in this study mean that even in clinical situations where atomoxetine plasma levels cannot be measured, atomoxetine can be relatively safely increased to the above target dose (110).

### Adverse Reactions

Atomoxetine was generally well tolerated in children and adolescents with ADHD (1, 26, 33, 35, 54, 101, 111–116). This section focuses primarily on data from trials including common and rare adverse reactions.

#### Common Adverse Reactions

The most common adverse reactions of atomoxetine in children with ADHD include gastrointestinal symptoms, sleep disturbances (somnolence), cardiovascular adverse reactions and other general disorders (irritability, dizziness, fatigue, headache) (103, 117). These four aspects will be described in detail below.

Gastrointestinal adverse reactions are one of the commonest adverse reactions in children with atomoxetine therapy (101), including appetite decrease, abdominal pain, vomiting and dyspepsia (117). With regard to an efficacy and safety extrapolation analysis (58), decreased appetite was the most common TEAE in atomoxetine-treated children at ages 6 to 7 (*n* = 565). In addition, the analysis concluded that in 5-year-old patients with ADHD, atomoxetine may improve ADHD symptoms, with some mood-related adverse reactions occurring at a higher rate (58). In short-term clinical trials, atomoxetine was reported to be more likely to have gastrointestinal adverse events than placebo (101). A meta-analysis of 6- to 8-week RCTs showed a decrease in appetite in 18.1% of 507 atomoxetine-treated patients aged 6–11 years compared to 5.3% of 339 placebo-treated patients (*p* < 0.001) (57). However, the incidence of gastrointestinal adverse reactions appeared to be highest within the first 1–3 months of treatment, and thereafter tended to subside over time in children (111).

Several short-term trials reported decreased weight in children after 6–8 weeks of atomoxetine therapy (118–122). In a 6-week RCT of 72 children with ADHD (ages 6 to 16), 9.7% (7/72) of the observation group had a loss of body mass compared with 6.1% (2/33) of the placebo group (121). A meta-analysis showed that children with ADHD (6 to 11 years old) had an average weight loss of 0.58 kg after atomoxetine treatment for 6 to 8 weeks (*n* = 507) (57). However, Spencer et al. (123) followed up the relationship between atomoxetine treatment and body mass for 5 years, and found that the body mass of children with ADHD showed the largest decline after 15-month atomoxetine treatment, partially recovered after 24 months, and recovered to the standard body mass after 36 months. The effect on the growth rate may be temporary. With the prolongation of treatment time, the growth level and speed may be gradually corrected (71, 124). Reductions in growth appear to be reversible in the long term (101). It may be that weight loss are secondary to decreased appetite, which may itself be related to other adverse reactions such as nausea, vomiting, and/or upper abdominal pain. The decreases in growth after atomoxetine treatment may be associated with gastrointestinal adverse reactions, but the long-term treatment has little effect on the physical growth of children with ADHD (58, 101).

Atomoxetine may cause somnolence more often than insomnia (113). Somnolence is the most common sleep-related adverse reaction associated with atomoxetine compared to stimulants (114). Previous studies showed that atomoxetine therapy significantly reduced sleep structure and sleep-wake cycle problems probably on account of lack of dopaminergic effects and appeared to be superior to stimulants (115, 125). In a meta-analysis of double-blind RCTs of atomoxetine treatment in children with ADHD, the results demonstrated that compared with placebo treatment, children with atomoxetine treatment had no significant increase in the incidence of sleep-onset insomnia and rarely resulted in treatment interruption (54). Additionally, atomoxetine was related to shorter sleep-onset latencies, lower frequency of insomnia, higher frequency of somnolence and smaller effects on subjective measures of sleep compared to methylphenidate (116). Although both atomoxetine and methylphenidate decreased nighttime awakenings, atomoxetine treatment was correlated with more frequent night awakenings (57, 116, 126). Compared with placebo, children with atomoxetine therapy exhibited significantly higher rates of somnolence and headache, although there was no significant difference in rates of headache between children and adolescents (57). Irritability, dizziness, fatigue, and headache are common in ADHD children with atomoxetine therapy (103). It is not only parts of clinical presentation, but also adverse reactions of some standard medications. In short-term RCTs of ADHD medications, there was an increased risk of headache for atomoxetine treatment compared to placebo (103, 127). The comprehensive results showed a significant association between atomoxetine treatment and headache in children with ADHD (127).

A meta-analysis of 16 studies reported age-group differences in the tolerability profile of atomoxetine (71). Younger children (6–7 years old) were more likely to experience more impairing sedative effects or abdominal pain or upset with atomoxetine treatment compared with placebo. Older children (aged 8–12 years) reported feeling sleepy or tired, which were more often described as irritable. In both age groups, those taking atomoxetine were more likely to complain of a diminished appetite and increased blood pressure than those taking placebo (71).

Elevated blood pressure and heart rate have been reported in patients with atomoxetine therapy (101). However, the effects of most patients on the cardiovascular system have no clinical significance (101, 128, 129). Hammerness et al. (130) found a statistically significant increase in heart rate in 6- and 7-year-old children with ADHD who received atomoxetine compared with placebo (*p* < 0.001). A study in Italy observed that the average heart rate in children with ADHD increased by 2.93 beats /min after 6 months of atomoxetine treatment compared with the baseline value, with a statistically significant difference (*p* < 0.001), while there was no statistically significant difference between the heart rate at 12th and 24th month compared with the baseline value (*p* > 0.05). The results indicated that the effect of atomoxetine on heart rate tended to decrease with the prolongation of time (131). The US label and the European SPC describe reports of moderately elevated heart rate and blood pressure in children and adolescents with atomoxetine treatment, which may be more pronounced in 8–12 % of pediatric patients. However, in long-term analyses (≥ 2 years) of pediatric patients with atomoxetine therapy, mean changes in blood pressure were usually within age norms and were generally not clinically significant. Few patients discontinued due to cardiovascular adverse reactions (101, 129). Interestingly, in a recent *in vitro* study using patch-clamp technique to perform electrophysiological experiments at the heterogeneously expressed human heart muscle sodium channels (hNav1.5) in human embryonic kidney cells, atomoxetine blocked sodium channels in a state- and use-dependent manner (132). It means that atomoxetine potentially has antiarrhythmic properties and reveals stronger anti-arrhythmic than pro-arrhythmic properties (132).

#### Rare Adverse Reactions

Based on previous research reports, liver injuries, suicidality, aggression/hostility, psychosis, seizures and prolonged QT interval are uncommon or rare (101). Rarely, atomoxetine may be associated with severe liver injury (101). According to Bangs and colleagues, during the 4-year post-marketing period, when atomoxetine exposure had reached approximately 4.3 million patients, three spontaneously reported cases of reversible hepatitis have been identified with relation to atomoxetine therapy (133). Another study, using the Italian ADHD National Registry, found that two of 68 pediatric patients experienced severe hepatic events after receiving unspecified doses of atomoxetine for 7 months and 10 months. In both cases, liver enzyme levels returned to normal after discontinuing atomoxetine therapy (118). In the long-term study of Eli Lilly's clinical trials database, Donnelly et al. (111) reported that ≤ 2% of 704 children and adolescents who received atomoxetine showed potentially clinically significant hepatic changes. These findings were based on laboratory tests for hepatic enzymes, including alanine aminotransferase (ALT), aspartate aminotransferase (AST) and alkaline phosphatase, and total bilirubin (111). Crucially, no patients had both a clinically significant increase in bilirubin (> 1.5 x ULN) combined with a significant increase in ALT (3 x ULN), which is a marker for potentially significant hepatic damage (111).

When children were treated with atomoxetine, small changes, or no changes/no clinically significant changes occurred in electrocardiograms (ECGs), including mean corrected QT (QTc) interval. Furthermore, atomoxetine is generally not associated with QT interval prolongation (134). There is a small but potential risk of QT interval prolongation (101).

The US prescribing information carries a black box warning on an elevated risk of suicidal ideation in children and adolescents with atomoxetine therapy (135) and it appears to be more common among atomoxetine than placebo recipients. A long-term (3–4 years) study found that among 714 children, 508 of them took atomoxetine for more than 4 years, and the incidence of suicidal ideation and behaviors was 0.8% (4/508) (111). Despite the black box warning on atomoxetine related suicidal ideation, no evidence existed for an increased rate of suicide related events associated with the use of atomoxetine in the register based longitudinal study (136). Although it cannot be ruled out that atomoxetine may increase the risk of suicide, there are other factors, such as depression and antisocial behaviors, which often comorbid ADHD, that may also lead to suicide in children (137).

With regard to aggression and hostility, these behaviors are reported more frequently but not statistically significantly different with atomoxetine compared with placebo (54, 111, 138), but the data suggest that there may be no association between atomoxetine therapy and increased risk of aggression/hostility, and more data are required to draw a firm conclusion. Moreover, psychosis including bipolar affective disorder (BPD) appear to be uncommon in atomoxetine-treated children with ADHD.

## Conclusion

Atomoxetine, the first non-stimulant drug to treat ADHD, is a selective NRI (28, 32, 35). It highly selectively binds to the presynaptic membrane NE reuptake transporter and inhibits NE reuptake, but has low affinity with other neurotransmitters (28, 31–34). Therefore, it can increase the level of NE in the synaptic cleft and dopamine level in the PFC, enhance children's memory and attention, and significantly improve ADHD symptoms (34); However, dopamine level in nucleus accumbens will not be increased, so it will not cause abuse or addiction, and there is no potential risk of abuse and addiction (32); At the same time, atomoxetine does not increase dopamine level in the striatum, so it will not induce tic symptoms or increase dyspraxia (27, 32, 139). There are few adverse reactions and high safety, which has been widely used at present (26, 35, 54, 111, 117).

In addition, the metabolic half-life of atomoxetine in the central nervous system is long, and the inhibitory effect of once a daily on NET can be lasted up to 24 h, so as to control the symptoms of ADHD patients in a sustained and stable way (31). Therefore, atomoxetine has a better effect on the long-term treatment of ADHD and patients with psychiatric symptoms (35, 69). In addition to improving the core symptoms of ADHD, atomoxetine can also improve multiple functions of children with long-term treatment, including learning function, peer relationship, family relationship, cognitive function, executive function, social function and so on (8, 140–143). Some studies have shown that common comorbidities (such as ODD, anxiety disorder, TD and ASD) in ADHD patients are unaffected or improved after atomoxetine therapy (16, 27, 35, 72, 73). In children with ADHD combined with transient tic disorder, atomoxetine therapy will not affect the secretion of dopamine and 5-hydroxytryptamine receptors in the body, effectively avoiding the aggravation of tic disorder and having high bioavailability (144). Atomoxetine may also be the first-line treatment for patients with comorbid tics. Actually, atomoxetine is recommended as the first-line treatment for ADHD patients with comorbid tic disorder and comorbid anxiety disorder in the latest Chinese guideline revised in 2015 by the Chinese Medical Association.

In the process of treatment, clinicians should pay special attention to controlling the dose of atomoxetine. Generally, oral administration of atomoxetine to children can achieve better results. However, due to the relatively active or deficient CYP2D6 enzyme in some children, the absorption of atomoxetine will be affected to a certain extent (29, 30). Therefore, the rate of improvement of clinical symptoms and therapeutic effects of children after atomoxetine therapy vary from patient to patient. For further treatment, timely monitoring and individualized medication are required.

The previous mention of atomoxetine being more effective in older children than in younger children raises some thoughts. Preschool children are not fully developed. The proportion of organs to body weight, water content, plasma protein concentration, acid-base balance and other physiological conditions are changing, thereby changing the distribution and penetration of atomoxetine. Atomoxetine is excreted mainly in urine and a small amount in feces (145). The glomerular filtration rate is higher during childhood, thereby influencing the excretion. *CYP2D6* genotype may be irrelevant. Atomoxetine is primarily metabolized by CYP2D6 (29, 30), but this enzyme is thought to develop very rapidly early and reach adult capacity at several months of age (58). Therefore, it is expected that there will be no difference in CYP2D6 activity and function in patients aged 4–5 years compared with the older children (58). In addition, the younger children are more likely to suffer acute epigastric pain and vomiting than older children, which may cause loss and fail to fully absorb atomoxetine. Older children reported feeling sleepy or tired (52, 71). In a study of patients aged 6–18 years in South Korea, the 10–15 age group had lower adherence than the 16–18 age groups (146). The high school students are under substantial academic pressures and great expectations from their parents, especially regarding university entry (146). This finding may explain why high school students exhibited better adherence compared with elementary and middle school students. Children with comorbidities were more likely to have higher adherence (147–149). The comorbid conditions of children led to the parents' diligence in controlling symptoms (147). However, younger children may not have accurate diagnosis of comorbidities. More studies to support this suggestion are needed in the future. Atomoxetine currently has three dosage forms: tablet, capsule, and oral liquid (145, 150). The younger children have difficulty swallowing tablets and capsules, and oral liquid has a poor taste, so it is difficult to swallow completely, which will have a certain effect on the efficacy. Last but not least, younger children are too young and immature to understand care from parents or physicians during atomoxetine therapy. They may avoid taking atomoxetine or not take it as prescribed.

In this review, the safety of atomoxetine was described from two aspects: common adverse reactions and rare adverse reactions. The most common adverse reactions including gastrointestinal adverse reactions and cardiovascular related adverse reactions may be related to the inhibition of NE by atomoxetine (111, 124, 151). And the most common adverse reactions usually appear in the first few months of medication. With the extension of treatment time, its adverse reactions gradually decrease and can be gradually tolerated. The occurrence of prolonged QT interval may be associated with CYP2D6, but this enzyme may not be related to liver function-related adverse reactions (151, 152). The adverse reactions of atomoxetine were generally mild or moderate, and severe adverse events were quite rare.

For ADHD children with atomoxetine therapy, adverse reactions should be evaluated and rational drug use should be guided. At the same time, parents of children with ADHD should be informed of the medication, understanding the common adverse reactions and countermeasures, so as to improve the medication compliance of children with ADHD. In summary, the application of atomoxetine in the clinical treatment of children with ADHD can significantly improve their learning ability and clinical symptoms.

## Author Contributions

FC and Q-QL: conceptualization. DF and D-DW: writing—original draft preparation. DF, D-DW, H-LG, and FC: writing—review and editing. Y-HH, YX, XJ, and W-RF: performed the reference collection and summary. Y-ML and JX: critical review. FC and H-LG: funding acquisition. All authors have read and agreed to the published version of the manuscript.

## Funding

This research was supported by the Specially Appointed Medical Expert Project of the Jiangsu Commission of Health (2019) and Special Fund for Clinical Research of the Wu Jieping Medical Foundation (320.6750.2020-04-07). This study was also supported by the Scientific Research Support Foundation for Top Young Scholars at the Children's Hospital of Nanjing Medical University (2020).

## Conflict of Interest

The authors declare that the research was conducted in the absence of any commercial or financial relationships that could be construed as a potential conflict of interest.

## Publisher's Note

All claims expressed in this article are solely those of the authors and do not necessarily represent those of their affiliated organizations, or those of the publisher, the editors and the reviewers. Any product that may be evaluated in this article, or claim that may be made by its manufacturer, is not guaranteed or endorsed by the publisher.

## Abbreviations

NE: Norepinephrine
ADHD: Attention deficit/hyperactivity disorder
NET: Norepinephrine transporter
PFC: Prefrontal cortex
CYP2D6: Cytochrome P450 2D6
MRI: Magnetic resonance imaging
fMRI: Functional magnetic resonance imaging
IFC: Inferior frontal cortex
DLPFC: Dorsolateral prefrontal cortex
DMN: Default mode network
FDA: Food and Drug Administration
NRI: Norepinephrine reuptake inhibitor
ODD: Oppositional defiant disorder
ASD: Autism spectrum disorder
TD: Tics disorders
TS: Tourette syndrome
MOA: Mechanism of action
SNRI: Selective norepinephrine reuptake inhibitor
FALFF: Fractional amplitude of low-frequency fluctuations
RS-fMRI: Resting-state functional magnetic resonance imaging
PET: Positron emission tomography
LTP: Long-term potentiation
RCT: Randomized controlled trial
ADHD-RS: ADHD-Rating Scale
OROS MPH: Osmotic release oral system methylphenidate
ADHD – RS – IV Parent: Inv: ADHD Rating Scale-IV Parent Version, Investigator Administered
LD: Learning disorders
DBDs: Disruptive behavior disorders
CD: Conduct disorder
MDD: Major depressive disorder
CDRS-R: Children's Depression Rating Scale-Revised
CGI-Tic: Clinical Global Impressions Tic
Neuro-S: Neurologic Severity
YGTSS: Yale Global Tic Severity Scale total score
TSSR: Tic Symptom Self-Report total score
RD: Reading disorder
ABC-H: Aberrant Behavior Checklist – Hyperactivity
SPC: Summary of Product Characteristics
CPIC: Clinical Pharmacogenetic Implementation Consortium
EM: Extensive metabolizer
PM: Poor metabolizers
IM: Intermediate metabolizer
UM: Ultrarapid metabolizer
CGI-P: Conners' Global Index-Parent Version
TEAEs: Treatment-emergent adverse events
hNav1.5: Heart muscle sodium channels
ALT: Alanine aminotransferase
AST: Aspartate aminotransferase
ECGs: Electrocardiograms
QTc: Corrected QT
BPD: Bipolar affective disorder
ADHD-RS-IV: Attention-Deficit/Hyperactivity Disorder Rating Scale-IV
PARS: Pediatric Anxiety Rating Scale
CGI: Clinical Global Impressions
CTRS-R:S: Conners Teacher Rating Scale-Revised: Short Form.
